# First direct measurements of behavioural responses by Cuvier's beaked whales to mid-frequency active sonar

**DOI:** 10.1098/rsbl.2013.0223

**Published:** 2013-08-23

**Authors:** Stacy L. DeRuiter, Brandon L. Southall, John Calambokidis, Walter M. X. Zimmer, Dinara Sadykova, Erin A. Falcone, Ari S. Friedlaender, John E. Joseph, David Moretti, Gregory S. Schorr, Len Thomas, Peter L. Tyack

**Affiliations:** 1 Centre for Research into Ecological and Environmental Modelling, Scottish Oceans Institute, University of St Andrews, St Andrews, UK; 2 School of Biology and Sea Mammal Research Unit, Scottish Oceans Institute, University of St Andrews, St Andrews, UK; 3 Southall Environmental Associates Inc., Aptos, CA, USA; 4 Long Marine Laboratory, University of California, Santa Cruz, CA, USA; 5 Nicholas School of the Environment, Duke University, Beaufort, NC, USA; 6 Cascadia Research Collective, Olympia, WA, USA; 7 Centre for Maritime Research and Experimentation (STO-CMRE), NATO Science and Technology Organisation, La Spezia, Italy; 8 Department of Oceanography, Naval Postgraduate School, Monterey, CA, USA; 9 Naval Undersea Warfare Center, Newport, RI, USA

**Keywords:** acoustic disturbance, avoidance response, anthropogenic noise, mid-frequency active sonar, military, *Ziphius cavirostris*

## Abstract

Most marine mammal­ strandings coincident with naval sonar exercises have involved Cuvier's beaked whales (*Ziphius cavirostris*). We recorded animal movement and acoustic data on two tagged *Ziphius* and obtained the first direct measurements of behavioural responses of this species to mid-frequency active (MFA) sonar signals. Each recording included a 30-min playback (one 1.6-s simulated MFA sonar signal repeated every 25 s); one whale was also incidentally exposed to MFA sonar from distant naval exercises. Whales responded strongly to playbacks at low received levels (RLs; 89–127 dB re 1 µPa): after ceasing normal fluking and echolocation, they swam rapidly, silently away, extending both dive duration and subsequent non-foraging interval. Distant sonar exercises (78–106 dB re 1 µPa) did not elicit such responses, suggesting that context may moderate reactions. The observed responses to playback occurred at RLs well below current regulatory thresholds; equivalent responses to operational sonars could elevate stranding risk and reduce foraging efficiency.

## 1. Introduction

Unusual mass strandings of cetaceans, especially beaked whales, have been associated with the operation of military mid-frequency active (MFA) sonars; these sometimes fatal events have raised serious concern about impacts of sonar and other anthropogenic sounds on whales [1–3]. Behavioural responses to MFA sonar are thought to play an important role in the series of events that leads to such strandings [1]. An on-going series of controlled exposure experiments (CEEs) and opportunistic studies have begun to provide data on behavioural responses to MFA sonar by species including Blainville's beaked whales (*Mesoplodon densirostris*; [4]). Cuvier's beaked whales (*Ziphius cavirostris* Cuvier) make up the majority of fatalities in MFA-associated strandings [5], and *Ziphius* abundance along the US west coast is apparently declining [6], but until now, direct measurements of *Ziphius* behavioural responses to MFA sonar have not been described.

## 2. Material and methods

**Figure 1.**
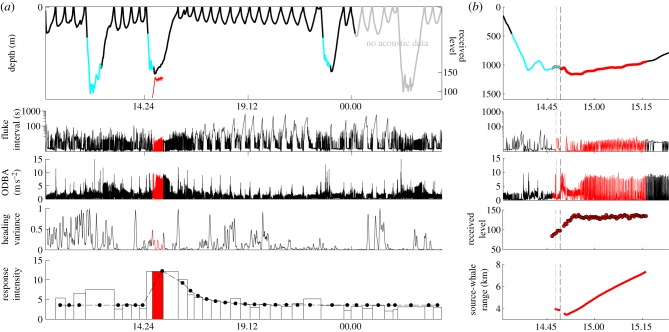
DTAG data from the *Ziphius* tagged in 2010, which underwent controlled exposure to simulated MFA sonar sounds. (*a*) Dive profile, with periods of echolocation clicking in cyan; time between fluke-strokes [10]; ODBA [12]; circular variance [13] of the animal's heading and the RI metric. For RI, boxes are observed data; dotted line with filled circles is fitted model output. (*b*) Zoomed view of the dive profile, fluke interval, ODBA, received MFA sonar level (dB re 1 µPa rms) and source-whale range. Grey vertical lines indicate the time of cessation of normal fluking in response to the sonar, and black lines the time when the strong avoidance response began. Throughout, pre- and post-exposure periods are in black, and controlled exposure periods in red.

**Figure 2.**
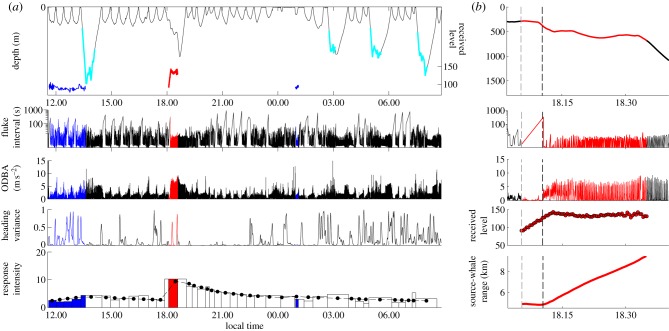
DTAG data from the *Ziphius* tagged in 2011, which underwent controlled exposure to simulated MFA sonar sounds and incidental exposure to naval MFA sonar. Figure layout and colour- and symbol-coding are the same as figure 1, but with blue traces for incidental exposure periods.

We present results from two Cuvier's beaked whales that were exposed to simulated MFA sonar during the Southern California Behavioural Response Study [7] in 2010–2011. The 2011 whale was also incidentally exposed to MFA sonar from a distant naval exercise. The whales were tagged with DTAGs ([8]; table 1), which recorded acoustic data (stereo, 16 bits, greater than or equal to 192 kHz sampling rate) and animal movement data (greater than or equal to 50 Hz sampling rate from tri-axial accelerometers and magnetometers and a pressure sensor, down-sampled to 5 Hz for analysis). To characterize baseline *Ziphius* behaviour, we used DTAG data from 13 whales in the Mediterranean Sea [9,10] and time-depth recorder data (mk9, Wildlife Computers, Redmond, WA, USA) from two whales in Hawai‘i ([11]; table 1). For each dive exceeding 50 m, we calculated: dive duration, maximum depth, duration and rate of descent and ascent, and time from surfacing until the next dive. For DTAG data only, we also calculated: duration of echolocation click production, time from surfacing until next echolocation click, average fluke-stroke rate [10], average overall dynamic body acceleration (ODBA; [12]), circular variance in heading [13] and source-whale range during exposures (figures 1 and 2; electronic supplementary material; data deposited in Dryad repository [14]).

**Table 1.** Details of tag deployments on *Ziphius cavirostris*.

tag ID	date and local time	tagging location (° N, ° E)	region	acoustic exposure	tag recording duration (h)	no. dives
zc03_260a	17 Sep 2003 17.48.03	44.1238, 8.8520	Mediterranean Sea	none	2.8	5
zc03_263a	20 Sep 2003 15.24.28	44.0960, 8.5897	Mediterranean Sea	none	15.6	19
zc04_160a	8 June 2004 16.44.23	44.0354, 8.7671	Mediterranean Sea	none	5.6	8
zc04_161a	9 June 2004 12.58.05	44.1107, 8.5819	Mediterranean Sea	none	8.9	18
zc04_161b	9 June 2004 14.07.37	44.0853, 8.5622	Mediterranean Sea	none	15.7	32
zc04_175a	23 June 2004 16.28.52	44.1117, 8.6856	Mediterranean Sea	none	7.5	6
zc04_179a	23 June 2004 16.28.52	44.1576, 8.7039	Mediterranean Sea	none	3.8	4
zc05_167a	16 June 2005 16.12.27	44.1568, 8.8253	Mediterranean Sea	none	7.6	15
zc05_170a	19 June 2005 15.24.13	44.1638, 8.7316	Mediterranean Sea	none	11.9	17
zc06_204a	23 July 2006 12.21.27	43.8190, 8.7255	Mediterranean Sea	none	6.2	6
zc06_205a	24 July 2006 13.57.43	43.7680, 8.7460	Mediterranean Sea	none	13.5	34
zc08_164a	12 June 2008 18.13.48	35.9321, −3.2773	Mediterranean Sea	none	16.2	25
zc10_272a	29 Sep 2010 09.49.43	32.8066, −119.0153	Southern California Bight	MFA	18.3	33
zc11_267a	24 Sep 2011 08.49.56	33.5105, −119.2806	Southern California Bight	MFA	21.3	39
zc12_169a	17 June 2012 17.49.40	43.7383, −8.4742	Mediterranean Sea	none	14.3	16
Nov. 2004 (mk9)	28 Nov 2004 13.02	19.32219, −156.04654	West of Hawai‘i	none	9.8	12
Nov. 2006 (mk9)	30 Nov 2006 09.41	19.44436, −156.05162	West of Hawai‘i	none	34.1	38
totals						
control (DTAG)					129.6	205
control (mk9)					43.9	50
exposed (DTAG)					39.6	72
all					213.1	327

CEE methods are described elsewhere [7]. After a 4.9 h (2010) or 9.3 h (2011) pre-exposure period, each whale experienced a 30-min controlled exposure from a stationary sound source 3.4–9.5 km away. During this period, the source transmitted a 1.6 s simulated MFA sonar signal every 25 s. The initial source level of 160 dB re 1 μPa-m was increased (‘ramped up’) by 3 dB per transmission to a maximum of 210 dB re 1 µPa-m. Incidental MFA sonar from a distant naval exercise was detected on the tag before and after the 2011 CEE. Based on US Navy logs, the ships were approximately 118 km away. RLs of MFA sonar signals from controlled and incidental exposures (calculated as in [4]) were 84–144 and 78–106 dB re 1 μPa root mean squared (rms), respectively (figures 1 and 2). (All RLs reported hereafter are in dB re 1 μPa rms)

Whale responses to MFA sonar sounds were scored according to a qualitative response severity scale [15]. To quantify overall response intensity (RI), we calculated a Mahalanobis distance-based RI metric that summarizes all DTAG dive parameters and quantifies how much each dive differs from the average baseline shallow or foraging dive. We modelled RI as a function of RL, source-whale range (2011 only, since 2010 had only one exposure dive), and time since sonar exposure (figures 1 and 2). We compared the full models with a nested set of models with fewer covariates using Akaike's information criterion (AIC; see the electronic supplemental materials for statistical details).

## 3. Results

Both whales showed clear responses to the CEEs, escalating from initial moderate orientation changes (corresponding to a score of 2 on the severity scale [15]) to a strong avoidance response sustained beyond the end of the exposure (severity score 8 [15]); however, the 2011 whale did not respond similarly to incidental naval sonar exposures (figures 1 and 2). When CEEs began, both whales stopped fluking, perhaps to monitor the sound and prepare to respond (2010: three 15–23 s pauses at RL 89–97 dB; 2011: 320 s pause at RL 90 dB; figures 1 and 2). In 2010, when RL reached 98 dB, the whale ceased its echolocation click production, interrupting foraging. It initiated an avoidance response that included energetic fluking, swimming away from the source at 2.6 m s^−1^, extended dive duration with an unusually slow ascent and a long (6.6 h) post-exposure inter-deep-dive interval (figure 1). The whale continued this strong and sustained avoidance (with rapid fluking, high ODBA and minimal heading variance) until about 1.6 h post-exposure (figure 1). In the 2011 CEE, when RL reached 127 dB, the whale initiated a similar avoidance response, including energetic fluking, swimming away from the source at 3.1 m s^−1^ and a long (7.6 h) post-exposure inter-deep-dive interval (figure 2; electronic supplementary material). This avoidance response lasted at least 1.7 h post-exposure (figure 2). The 2011 whale did not echolocate during the CEE dive, making the dive difficult to classify. It matches foraging dives in duration and depth but not dive shape and is unusually long and deep for a silent dive (figure 2; electronic supplementary material). By contrast, shallow dives coinciding with the distant naval exercise were similar to control shallow dives (figures 1 and 2; electronic supplementary material).

Statistical modelling of RI showed full models to be far superior to all reduced models (*Δ*AIC ≥ 28.5), suggesting that both RL and source-whale range influence response. RI remained elevated after exposure for 2.7 and 3.8 h in 2010 and 2011, respectively (figures 1 and 2).

## 4. Discussion

We observed intense, consistent, long-lasting responses by two *Ziphius* to simulated MFA sonar at short ranges. However, MFA sonar from a distant naval exercise did not elicit a similar response, even at comparable RLs. Source-whale range may therefore moderate a level-driven response to sonar, as may other factors (e.g. ramp-up; behavioural/environmental context). We note that *Ziphius* resident in the study area probably have prior experience with MFA sonar, as naval operations routinely occur in the region [16]. This complexity should be considered carefully in interpreting these results and planning future studies.

Our results extend previous findings on beaked whale noise responses [4,9,17], addressing in detail the key case of *Ziphius* and MFA sonar. Response of one *Ziphius* to ship noise (136 dB maximum received level) included cessation of echolocation, as in our MFA sonar CEEs, but in contrast, dive duration and inter-deep-dive interval were short (see the electronic supplementary material; [9]). Acoustic recordings of Blainville's beaked whales during ship noise exposure were consistent with directed travel [17]. Early cessation of clicking and horizontal avoidance are also components of MFA sonar response by Blainville's beaked whales [4]. Cessation of echolocation clicks was thus a common response to acoustic disturbance in beaked whales studied to date, but sonar avoidance responses were stronger and more prolonged than responses to vessel noise.

The silent, underwater avoidance reaction of *Ziphius* to MFA sonar is consistent with their cryptic behaviour: they spend minimal time at the surface, rarely produce sounds other than echolocation clicks and click only at depths exceeding 200 m [10]. The observed responses included vigorous swimming and extended time without echolocation-based foraging, imposing a net energetic cost that (if repeated) could reduce individual fitness. Rapid, directed swimming could increase stranding risk, particularly if it occurs near shore [5]. Our results are inconsistent with the hypothesis that unusually rapid ascents cause emboli found in beaked whales from sonar-related strandings [3]. However, the dive profile and swim-speed alterations we observed might still affect dive metabolism, perhaps reducing capacity to control perfusion of tissues with inert gases, and increasing the risk of gas-bubble lesions during decompression [18].

Our results represent the first empirical demonstration of behavioural responses to MFA sonar by *Z. cavirostris*, the species that accounts for 69 per cent of recorded cetacean strandings associated with MFA sonar [5]. Although we observed strong responses to nearby, controlled exposures to simulated MFA sonar, we did not detect similar responses to distant, incidental exposure to naval sonar exercises at comparable RLs, which is an important consideration in the application of these data and should be explored in future experiments. However, we particularly emphasize that *Ziphius* initiated intense, sustained responses to controlled exposures at RLs of 89–127 dB. Current US management practices assume that significant behaviour disruption almost never occurs at exposure levels this low [19,20]. This study provides a much-needed scientific basis to inform decisions and reduce adverse effects of MFA sonar on beaked whales.
